# A case of corneal abscess following self-medication with topical dermatologic corticosteroids in a child

**DOI:** 10.1093/omcr/omaf064

**Published:** 2025-06-27

**Authors:** Firdaouss Essaddouqui Esslimani, Chaimae Khodriss, Soukaina Zefzoufi, Soukayna Kabbou, Meriem El Bahloul

**Affiliations:** Department of Ophthalmology, Faculty of Medicine and Pharmacy of Tangier, Abdelmak Essaadi University, Tangier, Morocco; Department of Ophthalmology, Faculty of Medicine and Pharmacy of Tangier, Abdelmak Essaadi University, Tangier, Morocco; Department of Ophthalmology, Faculty of Medicine and Pharmacy of Tangier, Abdelmak Essaadi University, Tangier, Morocco; Department of Dermatology, Faculty of Medicine and Pharmacy of Tangier, Abdelmak Essaadi University, Tangier, Morocco; Department of Ophthalmology, Faculty of Medicine and Pharmacy of Tangier, Abdelmak Essaadi University, Tangier, Morocco

**Keywords:** medical ophthalmology, dermatology, pediatrics, endocrinology and metabolism, pharmacology and pharmacy

## Abstract

Topical dermatologic corticosteroids (TDC), while essential in treating skin conditions, can cause severe complications when misused, particularly in children. This case study reports a 7-year-old girl with a history of long-term self-medication with 0.05% betamethasone for dermatological lesions, resulting in iatrogenic Cushing's syndrome and a severe corneal abscess. The child presented with visual loss, corneal infiltrates, and hypopyon. Ocular and biochemical tests confirmed the diagnosis. The management included antifungal and antibacterial treatments, intrastromal voriconazole injection, and amniotic membrane grafting. TDC were gradually discontinued, and tacrolimus was used for skin symptoms. After 3 months, visual acuity improved to 3/10, with residual corneal opacity. This case emphasizes the dangers of prolonged TDC use without supervision and the need for early intervention in ocular complications.

## Introduction

Topical dermatologic corticosteroids (TDC) are crucial for treating various skin conditions due to their anti-inflammatory effects. However, their misuse, particularly in children, can lead to significant systemic and local complications. Prolonged use of TDC like betamethasone, especially without medical supervision, can cause severe side effects, including iatrogenic Cushing's syndrome and ocular complications. This case study highlights the dangers of long-term self-medication, exemplified by a severe corneal abscess in a child. It emphasizes the importance of early diagnosis and proper treatment to prevent serious ocular complications, particularly with potent topical corticosteroids.

## Case presentation

A 7-year-old girl consulted our emergency department for a red, painful left eye with reduced visual acuity that had been progressing for 10 days. She had no history of surgical trauma or ocular disease. However, the family reported that the child had been self-medicating with an ointment containing betamethasone dipropionate 0.05% for over 5 years for generalized pruritic dermatological lesions, including on the face. The child also developed impaired concentration, which had a significant impact on her daily life, to the extent that she was forced to interrupt her studies. A neuropsychological evaluation did not reveal any clinically substantial disorder requiring specific treatment.

**Figure 1:**
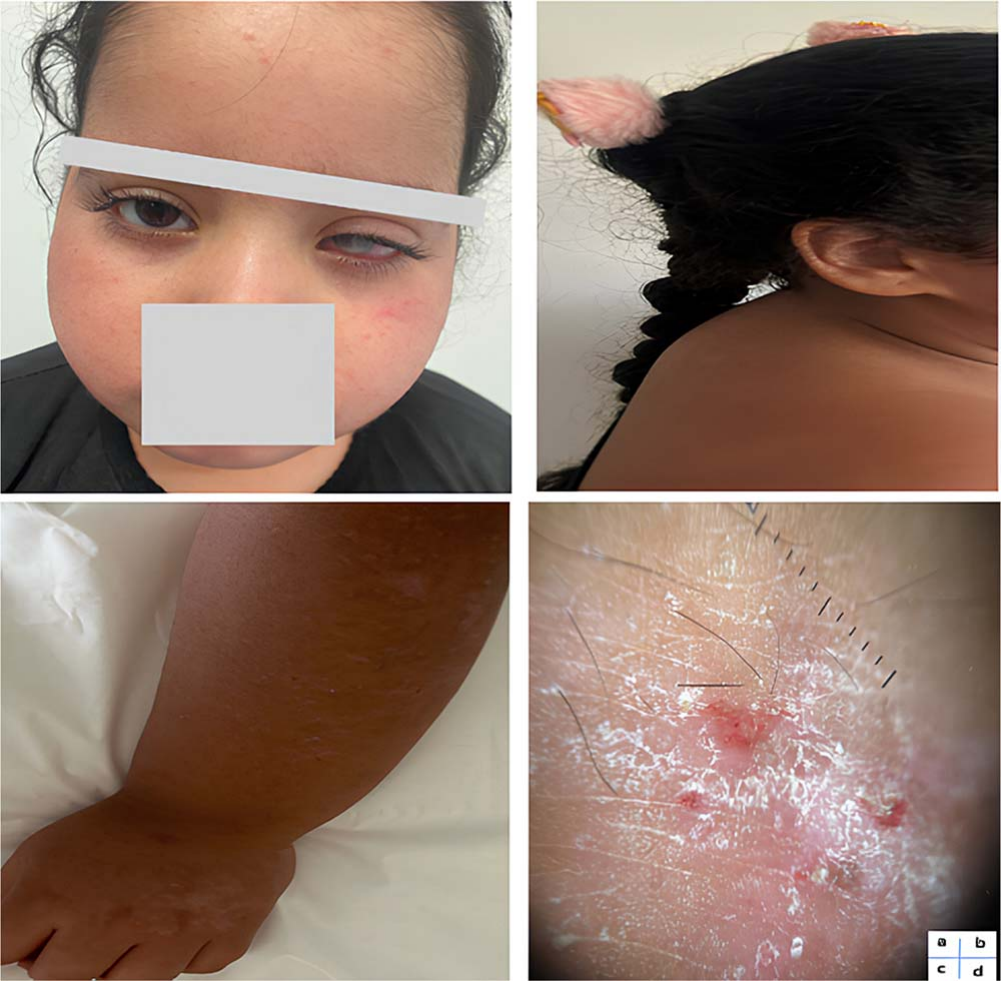
**a**: Moon face and periorbital dermatitis. **b**: Buffalo hump appearance. **c**: Skin pigmentation changes on hands and forearms. **d**: Dermoscopic appearance of an active dermatitis lesion.

General examination revealed a cushingoid appearance, with facies and trunk obesity, and a buffalo hump (Fig. 1a and b). This clinical profile led us to suggest Cushing's syndrome.

A dermatology opinion was taken for squamous erythematosus placards on the face, forearms, and legs. The patient was diagnosed as atopic dermatitis.

**Figure 2:**
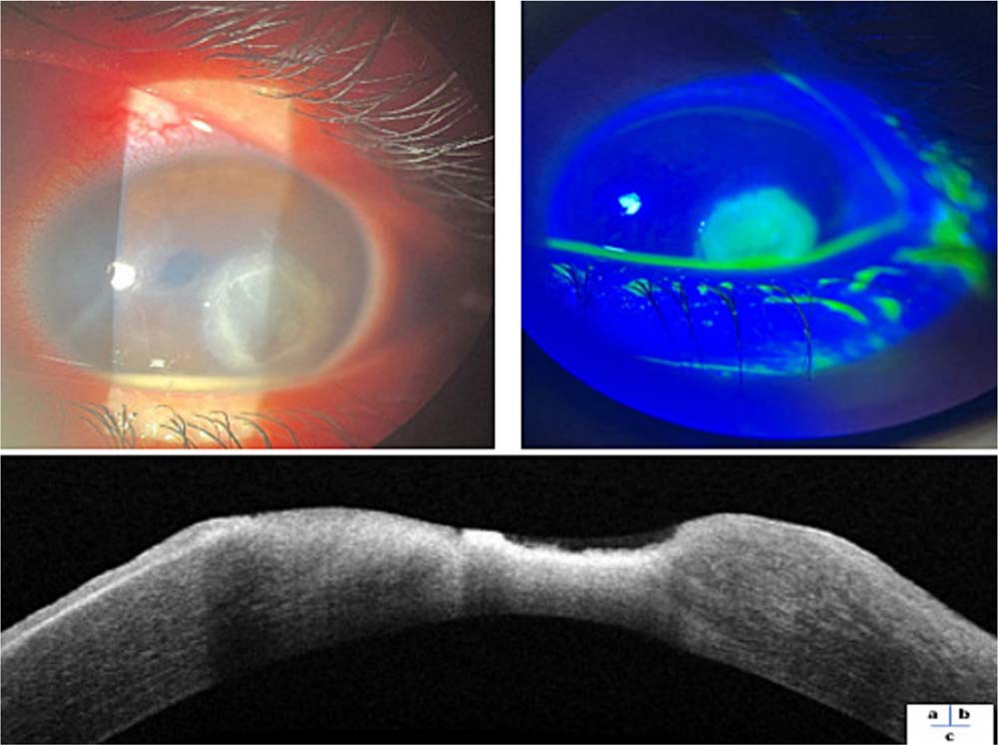
**a**: Slit lamp photograph of corneal ulcer with hypopyon. **b**: Positive fluorescein test. **c**: Anterior segment OCT showed significant irregular stromal loss.

Ophthalmological examination of the left eye revealed visual acuity limited to positive light perception with a para-axial epithelial-stromal corneal infiltrate measuring 4.8 mm x 3.5 mm with raised blurred margins and central thinning (Fig. 2a). The fluorescein test was positive (Fig. 2b) and a hypopyon was present in the anterior chamber. Consequently, neither the lens nor the fundus could be visualized. Ocular ultrasound revealed no evidence of vitreous locules or retinal detachment. Additionally, no foreign body was detected upon eversion of the eyelid. A slit lamp examination of the right eye was unremarkable. Anterior segment OCT showed significant irregular stromal loss (Fig. 2c).

An endocrinological consultation was carried out. Based on their clinical assessment, the endocrinologists ordered biochemical tests to assess hormone function. Results revealed low morning cortisol at 7 nmol/l (normal range: 200–750 nmol/l) and corticotropic hormone (ACTH) deficiency at 0.3 pmol/l (normal range: 2.2–13.2 pmol/l). The urinary steroid profile showed very low steroid metabolites, while urinary free cortisol was elevated to 1500 nmol/day (normal range: < 150 nmol/day). led to the diagnosis of iatrogenic Cushing's syndrome.

After hospitalization, a corneal swab was taken, and the culture came back negative. Topical antifungal treatment with fortified eye drops of voriconazole (10 mg/ml) and oral fluconazole (5 mg/kg/day) were prescribed because of the strong suspicion of a fungal origin of the corneal abscess.

Considering the severity of the clinical aspect of our patient, empirical treatment with broad-spectrum antibiotics was included, including fortified eye drops of vancomycin (50 mg/ml) and ceftazidime (20 mg/ml). Treatment was completed with eye drops of ciprofloxacin (0.3%), moxifloxacin (0.5%) and ciprofloxacin (0.3%) ointment, pupillary dilation with tropicamide 0.25% was added.

Antiviral treatment with oral acyclovir (20 mg/Kg/day) five times per day was added to cover any viral component of the corneal infection. To improve the healing and protection of the cornea, topical bio-protectors combining trehalose and hyaluronic acid were administered.

**Figure 3:**
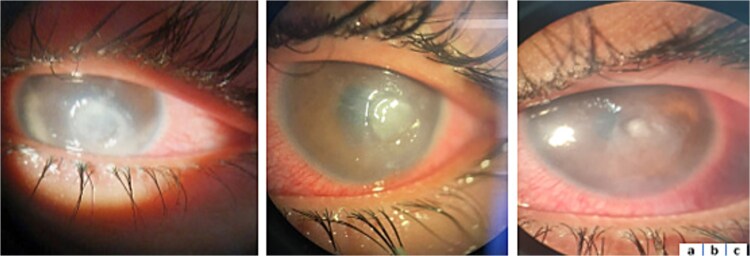
Clinical evolution of corneal abscess under medical treatment. **a**: Day 7. **b**: Day 14. **c**: Day 21.

Intrastromal injection of voriconazole 0.05% in five barrier zones surrounding the corneal infiltrate was performed. The evolution was characterized by improved functional signs and reduced abscess size (Fig. 3).

**Figure 4:**
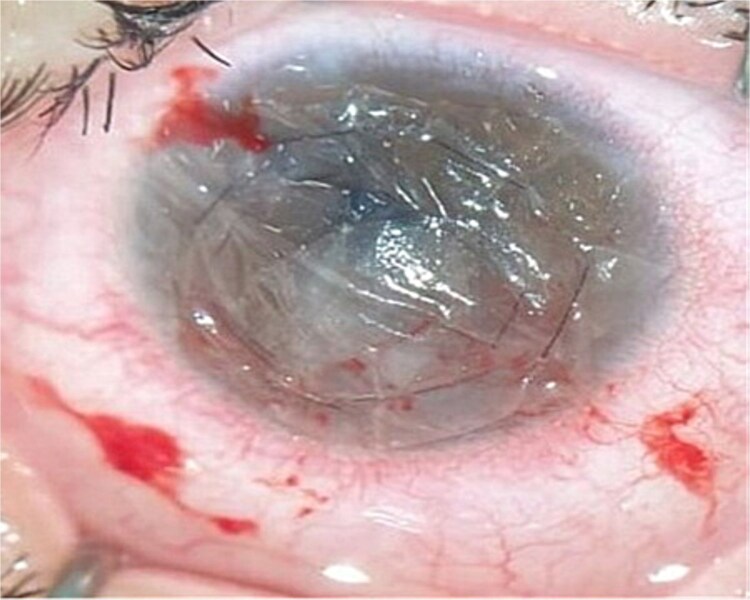
After amniotic membrane grafting technique (overlay-inlay).

In addition, an amniotic membrane graft was placed in the bottom of the ulcer (inlay-overlay) after infection control to seal the pre-perforative site (Fig. 4.)

TDC applications were progressively reduced, and topical tacrolimus 0.03% ointment was applied to control cutaneous symptoms. No specific hormonal dysfunction was identified during the endocrinological follow-up, and plasma ACTH and cortisol levels returned to normal limits.

**Figure 5:**
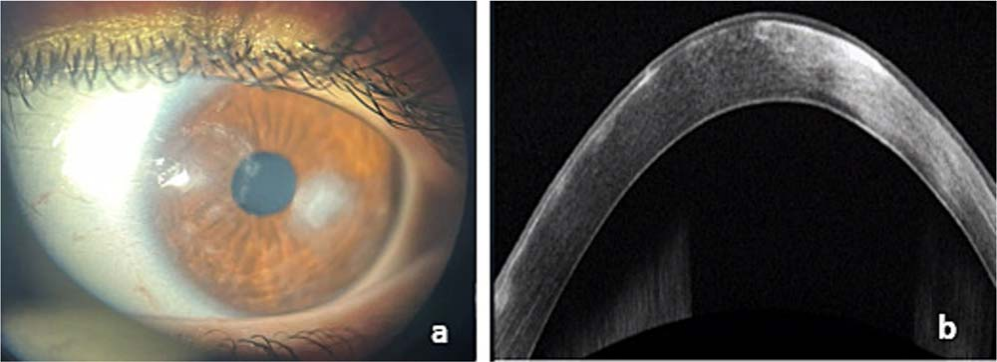
Progression after 3 months. **a**: Regression of the corneal abscess with residual corneal opacity. **b**: OCT image showing stromal hyperreflectivity accompanied by slight corneal thinning, associated with corneal opacity.

After 3 months of treatment and follow-up, the best-corrected visual acuity in the left eye was 3/10, and a residual corneal opacity persisted (Fig. 5).

## Discussion

Topical corticosteroids are known to be associated with various ocular complications, including cataracts, glaucomatous neuropathy, and opportunistic infections [1]. TDC-related complications are less commonly reported, but cases have linked corticosteroid creams on the periorbital region to the development of glaucoma [2, 3]. One case described cataracts, glaucoma, and femoral avascular necrosis following the use of moderately potent TDC for discoid eczema [4].

While ophthalmologists recognize infectious complications from topical ophthalmic steroids, those linked to TDC are rarer. *To our knowledge, this represents the first case documented in the literature of infectious keratitis in a child following prolonged and improper TDC use.*

The pathophysiology of ocular complications involves systemic absorption through the skin and local diffusion through the eyelids [5]. Steroids compromise ocular immunity, increasing vulnerability to infections. For instance, one patient on systemic corticosteroids developed bilateral herpetic keratitis [6], while another study found topical steroids were linked to more aggressive fungal keratitis [7]. Additionally, Candida endophthalmitis has been reported in patients on systemic corticosteroids [8, 9].

In our case, the patient's young age increased her risk of systemic reactions, aggravated by the daily and prolonged use of betamethasone dipropionate over large areas of her body, without clear dosing guidelines. In addition, the amount of corticosteroid was not applied in standardized units such as the Finger Tip Unit (FTU), which may have led to excessive and uncontrolled use. This high-potency corticosteroid, informally applied by the patient's mother, was combined with an impaired skin barrier due to atopic dermatitis, which probably resulted in increased systemic absorption and contributed to the development of infectious keratitis. Despite a negative corneal culture, the fungal origin of the abscess. Was suspected due to its clinical appearance and the state of immunosuppression. In addition, the patient presented with neuropsychological disturbances in the form of attention deficit disorder, which could be related to the prolonged use of corticosteroids, known for their neuropsychiatric effects.

## Conclusion

This case highlights the dangers of self-medication and improper use of TDC, particularly in children. It underscores the need for patient education on adhering to prescribed treatments and the importance of follow-up, especially with potent corticosteroids. Additionally, healthcare professionals, including dermatologists, ophthalmologists, and pharmacists, must be aware of the risks associated with topical corticosteroids. The case calls for improved collaboration and education to enhance visual health outcomes.
